# Fluid velocity based simulation of hydraulic fracture: a penny shaped model—part I: the numerical algorithm

**DOI:** 10.1007/s11012-018-0899-y

**Published:** 2018-10-22

**Authors:** Daniel Peck, Michal Wrobel, Monika Perkowska, Gennady Mishuris

**Affiliations:** 1 Aberystwyth University, Aberystwyth, UK; 2 AGH University of Science and Technology, Cracow, Poland; 3 EnginSoft S.p.A., Trento, Italy

**Keywords:** Penny-shaped crack, Hydraulic fracture, Universal algorithm, Power law fluid, Leak-off, Speed equation

## Abstract

In the first part of this paper, a universal fluid velocity based algorithm for simulating hydraulic fracture with leak-off, previously demonstrated for the PKN and KGD models, is extended to obtain solutions for a penny-shaped crack. The numerical scheme is capable of dealing with both the viscosity and toughness dominated regimes, with the fracture being driven by a power-law fluid. The computational approach utilizes two dependent variables; the fracture aperture and the reduced fluid velocity. The latter allows for the application of a local condition of the Stefan type (the speed equation) to trace the fracture front. The obtained numerical solutions are carefully tested using various methods, and are shown to achieve a high level of accuracy.

**Electronic supplementary material:**

The online version of this article (10.1007/s11012-018-0899-y) contains supplementary material, which is available to authorized users.

## Introduction

Hydraulic fracture (HF) is the phenomenon of a fluid driven crack propagating in a solid material. It can be encountered in various natural processes, such as subglacial drainage of water or during the extension of magmatic intrusions in the earth’s crust. Simultaneously the underlying physical mechanism is very important in numerous man-made activities. Hydrofracturing can appear as an unwanted and detrimental factor in underground $\hbox {CO}_2$ or waste repositories [1]. On the other hand, intentionally induced hydraulic fractures constitute the essence of fracking technology - a method used when stimulating unconventional hydrocarbon reservoirs [2] or for geothermal energy exploitation [3]. All of these applications create demand for a proper understanding and prediction of the process of hydraulic fracture.

As a result of the multiphysical nature of the underlying physical phenomenon and complex interactions between the component physical fields, the mathematical modeling of hydraulic fractures represents a significant challenge. The main difficulties arise due to: (1) strong non-linearities resulting from interaction between the solid and fluid phases, (2) singularities in the physical fields, (3) moving boundaries, (4) degeneration of the governing equations at the crack tip, (5) leak-off to the rock formation, (6) pronounced multiscaling effects, vii) complex geometry.

The first theoretical models of hydraulic fracture were created in 1950s (see for example [4] and [5]). Subsequent research led to the formulation of the so-called classic 1D models: PKN [6, 7], KGD (plane strain) [8, 9] and penny-shaped/radial [10]. Up to the 1980s these very simplified models were used to design and optimize the treatments used in HF. The increasing number and size of fracking installations, alongside the simultaneous advance in computational techniques, resulted in the formulation of more sophisticated and realistic models of HFs. A comprehensive review of the topic can be found in [11].

Though superseded in most practical applications, the classic 1D models remain a significant avenue of research into the fundamentals of HF. They enable one to investigate some inherent features of the underlying physical process, the mathematical structure of the solution, and finally to construct and validate computational algorithms. Substantial advances have been achieved in this area throughout the last 30 years by way of a cyclical revision of these classic formulations. It was not until 1985 [12] that the importance of the solution tip asymptotics was first noticed, specifically for the KGD and penny shaped cracks. The explicit form of the tip asymptotic solution for the PKN model was given in 1990 by *Kemp* [13]. Moreover, in this publication the author remarked, for the first time, that when properly posed the Nordgren’s model constitutes a Stefan-type problem and as such needs an additional boundary condition which equates the crack propagation speed with the velocity of the fluid front. However, this important idea was abandoned for the next 20 years until being rediscovered by *Linkov* [14] in 2011. The author proved that the general HF problem is ill-posed and proposed a regularization technique based on application of the aforementioned Stefan condition—called there the speed equation. The numerous investigations carried out since the beginning of the present century for the KGD [15–18] and penny-shaped models [19–21] have led to the importance of the problem’s multiscale character being recognized. It is now well understood that the global response of the fluid driven fracture is critically dependent on the interaction between competing physical processes at various temporal and spatial scales. Depending on the intensity of various energy dissipation mechanisms, as well as the fracturing fluid and solid material properties, the hydraulic fracture evolves in the parametric space encompassed by the limiting regimes: (1) viscosity dominated, (2) toughness dominated, (3) storage dominated, (4) leak-off dominated.

Bearing in mind the whole complexity of the problem, it still remains an extremely challenging task to deliver credible solutions which reflect all of the desired features. The relative simplicity of the classic 1D models means that they are well suited to the task of creating benchmarks, used when developing and verifying more advanced solutions and algorithms. For the KGD and PKN models one can find in the literature a number of credible results, including recently developed simple and accurate approximate solutions, that can be utilized for the aforementioned purposes [22–25]. A more complete review of recent benchmarks will be provided in part II of this paper.

The aim of the first paper is to meet the demand for benchmark solutions to the radial HF model and: (1) to deliver a dedicated computational scheme capable of obtaining highly accurate numerical solutions, (2) introduce purely analytical solutions to the problem obtained for a predefined non-zero leak-off function, (3) introduce and verify an alternative measure of the computational error, to use when no analytical solution is available.

To this end the self-similar formulation of the penny-shaped model will be analyzed. The numerical computations will be performed according to a modified form of the universal algorithm introduced in [24, 25]. It employs a mechanism of fracture front tracing, based on the speed equation approach [23], coupled with an extensive use of information on the crack tip asymptotics and regularization of the Tikhonov type (the technical details of both concepts can be found in [26, 27]). The modular architecture of the computational scheme facilitates its adaptation to the problem of radial HF.

It is worth stating that the second part of this paper will introduce simple to use semi-analytical approximations of numerical benchmark solutions obtained for the case of an impermeable solid, and comparisons with other available data will be performed. Both parts are written in such a way that they can be read as individual, independent papers (for a unified version of the text, see arXiv:1612.03307).

The paper is organized as follows. The basic system of equations describing the problem is given in Sect. 2. Next, normalization to the dimensionless form is carried out. In Sect. 3, comprehensive information about the solution asymptotics is presented, which is heavily utilized in the subsequent numerical implementation. New computational variables, the reduced fluid velocity and modified fluid pressure derivative, are introduced. The advantages of both are outlined, and the problem is reformulated in terms of the new variables. In Sect. 4 the governing system of equations is reduced to the time independent self-similar form. This formulation is used in Sect. 5 to construct the computational algorithm. The accuracy and efficiency of computations are examined against newly introduced analytical benchmark examples. Alternative error measures are proposed for the cases where no closed-form analytical solution is available. Section 6 contains the final discussion and conclusions. Some additional information concerning the limiting cases of Newtonian and perfectly plastic fluids is collected in the appendices.

## Problem formulation

Let us consider a 3D penny-shaped crack, defined in polar coordinates by the system $\{r,\theta ,z\}$, with associated crack dimensions $\{l(t),w(t)\}$ as the fracture radius and aperture respectively, noting that both are functions of time. The crack is driven by a point source located at the origin, which has a known pumping rate: $Q_0(t)$. The fluid’s rheological properties are described by the power-law model [28], as is common in the literature. The rational behind this choice is outlined in [25]. We have that, as the flow is axisymmetric, all variables will be independent of the angle $\theta$ and it is sufficient to analyse the problem for only $r\ge 0$.

The fluid mass balance equation is as follows:

1 $$\begin{aligned} \frac{\partial w}{\partial t} + \frac{1}{r} \frac{\partial }{\partial r}\left( r q \right) + q_l = 0 , \quad 0<r<l(t), \end{aligned}$$

where $q_l (r,t)$ is the fluid leak-off function, representing the volumetric fluid loss to the rock formation in the direction perpendicular to the crack surface per unit length of the fracture. We will assume it to be a predefined and smooth function which is bounded at the fracture tip.

Meanwhile, *q*(*r*, *t*) is the fluid flow rate inside the crack, given by the Poiseuille law:

2 $$\begin{aligned} q^n =-\frac{w^{2n+1}}{M^\prime }\frac{\partial p}{\partial r} , \end{aligned}$$

with *p*(*r*, *t*) being the net fluid pressure on the fracture walls (i.e. $p=p_f - \sigma _0$, where $p_f$ is the total pressure and $\sigma _0$ is the confining stress). The constant $M^\prime$ is the so-called modified fluid consistency index $M^\prime = 2^{n+1} (2n+1)^n / n^n M$, where *M* stands for the consistency index (relating the shear stress and strain rate) and $0\le n \le 1$ is the fluid behaviour index.

The non-local relationships between the fracture aperture and the pressure (elasticity equations) are as follows:

3 $$\begin{aligned} p(r,t) = \frac{E^\prime }{l(t)} {{\mathcal {A}}}[w](r,t) , \quad w(r,t) = \frac{l(t)}{E^\prime } {{\mathcal {A}}}^{-1}[p](r,t) , \end{aligned}$$

where $E^\prime =Y/(1-\nu ^2)$, with *Y* being the Young’s modulus and $\nu$ the Poisson ratio. The operator ${\mathcal {A}}$ and its inverse take the form:

4 $$\begin{aligned} {{\mathcal {A}}}[w]= & {} - \int _0^1 \frac{\partial w(\eta l(t), t)}{\partial \eta } M\left[ \frac{r}{l(t)},\eta \right] \, d\eta , \end{aligned}$$

5 $$\begin{aligned} {{\mathcal {A}}}^{-1} [p]= & {} \frac{8}{\pi } \int _{r/l(t)}^1 \frac{\xi }{\sqrt{\xi ^2 - (r/l(t))^2}} \int _0^1 \frac{\eta p(\eta \xi l(t),t)}{\sqrt{1-\eta ^2}} \, d\eta \, d\xi \nonumber \\\equiv & {} \frac{8}{\pi } \int _0^1 \eta p(\eta l(t), t) G\left[ \frac{r}{l(t)} , \eta \right] \, d\eta \, , \end{aligned}$$

with the pertinent kernels being:

6 $$M\left[ {\xi ,s} \right] = \frac{1}{{2\pi }}\left\{ {\begin{array}{*{20}l} {\frac{1}{\xi }K\left( {\frac{{s^{2} }}{{\xi ^{2} }}} \right) + \frac{\xi }{{s^{2} - \xi ^{2} }}E\left( {\frac{{s^{2} }}{{\xi ^{2} }}} \right),} \hfill & {\xi > s} \hfill \\ {\frac{s}{{s^{2} - \xi ^{2} }}E\left( {\frac{{\xi ^{2} }}{{s^{2} }}} \right),} \hfill & {s > \xi } \hfill \\ \end{array} } \right.$$

7 $$\begin{aligned} G(\xi , s)= & {} {\left\{ \begin{array}{ll} \frac{1}{\xi } F\left( {\arcsin \left( \sqrt{\frac{1-\xi ^2}{1-s^2}}\right) }\Big \vert { \frac{s^2}{\xi ^2}}\right) , \quad \xi>s \\ \frac{1}{s} F\left( {\arcsin \left( \sqrt{\frac{1-s^2}{1-\xi ^2}}\right) }\Big \vert { \frac{\xi ^2}{s^2} }\right), \quad s>\xi \end{array}\right. } \end{aligned}$$

*K*, *E* are the complete elliptic integrals of the first and second kinds respectively, and *F* is the incomplete elliptic integral of the first kind, given in [29].

These equations are supplemented by the boundary condition at $r=0$, which defines the intensity of the fluid source, $Q_0$:

8 $$\begin{aligned} \lim _{r \rightarrow 0} r q(r,t) = \frac{Q_0(t)}{2\pi } , \end{aligned}$$

the tip boundary conditions:

9 $$\begin{aligned} w(l(t),t)=0 , \quad q(l(t),t)=0 , \end{aligned}$$

and appropriate initial conditions describing the starting crack opening and length:

10 $$\begin{aligned} w(r,0) = w_* (r) , \quad l(0) = l_0 . \end{aligned}$$

Additionally, it is assumed that the crack is in continuous mobile equilibrium, and as such the classical crack propagation criterion of linear elastic fracture mechanics is imposed [30]:

11 $$\begin{aligned} K_I = K_{I c} , \end{aligned}$$

where $K_{I c}$ is the material toughness while $K_I$ is the stress intensity factor. The latter is computed according to the following formula [31]:

12 $$\begin{aligned} K_I (t) = \frac{2}{\sqrt{\pi l(t)}} \int _0^{l(t)} \frac{r p(r,t)}{\sqrt{l^2(t) - r^2}} \, dr . \end{aligned}$$

Throughout this paper we accept the convention that when $K_{Ic}=0$ the hydraulic fracture propagates in the viscosity dominated regime. Otherwise the crack evolves in the toughness dominated mode. Each of these two regimes is associated with qualitatively different tip asymptotics, which constitutes a singular perturbation problem as $K_{Ic} \rightarrow 0$, and leads to serious computational difficulties in the small toughness range.

Finally, noting (1) and (8), the global fluid balance equation is given by:

13 $$\begin{aligned} \int _0^{l(t)} r\left[ w(r,t) - w_0(r) \right] \, dr \, + \int _0^t \int _0^{l(t)} r q_l (r,\tau ) \, dr \, d\tau = \frac{1}{2\pi } \int _0^t Q_0 (\tau ) \, d\tau . \end{aligned}$$

The above set of equations and conditions represents the typically considered formulation for a penny-shaped hydraulic fracture [19].

In order to facilitate the analysis we shall utilize an additional dependent variable, *v*, which describes the average speed of fluid flow through the fracture cross-section [23]. It will be referenced to in the text as the fluid velocity (often referred to in the literature as the particle velocity, e.g. [24, 25]), and is defined as:

14 $$\begin{aligned} v(r,t) = \frac{q(r,t)}{w(r,t)} , \quad v^n (r,t) = -\frac{1}{M^\prime } w^{n+1} \frac{\partial p}{\partial r} . \end{aligned}$$

We assume that the leak-off $q_l$ is such that the fluid velocity is finite at the crack tip, meaning that *v* has the following property:

15 $$\begin{aligned} \lim _{r\rightarrow l(t)} v(r,t) = v_0 (t) <\infty . \end{aligned}$$

Additionally, given that the fracture apex coincides with the fluid front (there is no lag), and that the fluid leak-off at the fracture tip is bounded, the so-called *speed equation* [14] holds:

16 $$\begin{aligned} \frac{d l}{dt} =v_0(t) . \end{aligned}$$

This Stefan-type boundary condition constitutes an explicit method, as opposed to an implicit level-set method [32], and can be effectively used to construct a mechanism of fracture front tracing. The advantages of implementing such a condition have been shown in [24, 25].

### Problem normalization

In order to make the presentation clearer, we will assume that $0<n<1$ in the main body of the text. Any modification to the governing equations and numerical scheme in the limiting cases $n=0$ and $n=1$ are detailed in “Appendix 1”.

We normalize the problem by introducing the following dimensionless variables:

17 $$\begin{aligned} \begin{aligned} \tilde{r}&= \frac{r}{l(t)} , \quad \tilde{t}=\frac{t}{t_n^{1/n}}, \quad \tilde{w}(\tilde{r},\tilde{t})=\frac{w(r,t)}{l_*} , \quad L(\tilde{t})=\frac{l(t)}{l_*} , \quad \tilde{q}_l (\tilde{r}, \tilde{t}) = \frac{t_n^{1/n}}{l_*} q_l (r,t) ,&\\&\tilde{q}(\tilde{r},\tilde{t}) = \frac{t_n^{1/n}}{l_*^2} q(r,t) , \quad \tilde{Q}_0(\tilde{t})=\frac{t_n^{1/n}}{l_*^2 l(t)}Q_0(t), \quad \tilde{v}(\tilde{r},\tilde{t})=\frac{t_n^{1/n}}{l_*}v(r,t),&\\&\tilde{p}(\tilde{r},\tilde{t}) = \frac{t_n}{M^\prime } p(r,t) , \quad \tilde{K}_{Ic} = \frac{1}{E^\prime \sqrt{l_*}}K_{Ic} , \quad t_n=\frac{M^{\prime }}{E^\prime } ,&\end{aligned} \end{aligned}$$

where $\tilde{r}\in \left[ 0,1\right]$ and $l_*$ is chosen for convenience.

We note that such a normalization scheme has previously been used in [24, 25], and that it is not attributed to any particular influx regime or asymptotic behaviour of the solution.

Under normalization scheme (17), the continuity equation (1) can be rewritten in terms of the fluid velocity (14) to obtain:

18 $$\begin{aligned} \frac{\partial \tilde{w}}{\partial \tilde{t}} - \frac{L^\prime (\tilde{t})}{L(\tilde{t})} \tilde{r} \frac{\partial \tilde{w}}{\partial \tilde{r}} + \frac{1}{L(\tilde{t})\tilde{r}} \frac{\partial }{\partial \tilde{r}}\left( \tilde{r}\tilde{w}\tilde{v}\right) + \tilde{q}_l = 0 . \end{aligned}$$

The fluid velocity (2) is expressed as:

19 $$\begin{aligned} \tilde{v}= \left[ -\frac{\tilde{w}^{n+1}}{L(\tilde{t})}\frac{\partial \tilde{p}}{\partial \tilde{r}} \right] ^{\frac{1}{n}} , \end{aligned}$$

while the speed equation is now given by combining (14)–(16):

20 $$\begin{aligned} \tilde{v}_0 (\tilde{t}) = L'(\tilde{t}) = \left[ - \frac{\tilde{w}^{n+1}}{L(\tilde{t})}\frac{\partial \tilde{p}}{\partial \tilde{r}} \right] ^{\frac{1}{n}}_{\tilde{r}=1} < \infty . \end{aligned}$$

The global fluid balance equation (13) is transformed to:

21 $$\begin{aligned} \begin{aligned}&\int _0^1 \tilde{r} \left[ L^2(\tilde{t})\tilde{w}(\tilde{r},\tilde{t}) - L^2(0)\tilde{w}_0(\tilde{r}) \right] \, d\tilde{r} \quad+ \int _0^{\tilde{t}} \int _0^1 \tilde{r} L^2 (\tau ) \tilde{q}_l (\tilde{r},\tau ) \, d\tilde{r} \, d\tau&\\&\quad = \frac{1}{2\pi } \int _0^{\tilde{t}} L(\tau ) \tilde{Q}_0 (\tau ) \, d\tau .&\end{aligned} \end{aligned}$$

The notation for the elasticity Eqs. (3)–(5) takes the form:

22 $$\begin{aligned} \tilde{p}(\tilde{r},\tilde{t}) = \frac{1}{L(\tilde{t})} {{\mathcal {A}}} [\tilde{w}](\tilde{r},\tilde{t}),\quad \tilde{w}(\tilde{r},\tilde{t}) = L(\tilde{t}) {{\mathcal {A}}}^{-1} [\tilde{p}](\tilde{r},\tilde{t}), \end{aligned}$$

where the operators denote:

23 $$\begin{aligned} {{\mathcal {A}}}[\tilde{w}](\tilde{r},\tilde{t})= & {} -\int _0^1 \frac{\partial \tilde{w}(\eta ,\tilde{t})}{\partial \eta } M\left[ \tilde{r},\eta \right] \, d\eta , \end{aligned}$$

24 $$\begin{aligned} {{\mathcal {A}}}^{-1}[\tilde{p}](\tilde{r},\tilde{t})= & {} \frac{8}{\pi }\int _{\tilde{r}}^1 \frac{\xi }{\sqrt{\xi ^2 - \tilde{r}^2}} \int _0^1 \frac{\eta \tilde{p}(\eta \xi ,\tilde{t})}{\sqrt{1-\eta ^2}} \, d\eta \, d\xi . \end{aligned}$$

From definition (12) and the fracture propagation condition (11) we have that:

25 $$\begin{aligned} \tilde{K}_I = \tilde{K}_{Ic} = \, \frac{2}{\sqrt{\pi }} \sqrt{L(\tilde{t})} \int _0^1 \frac{\tilde{r} \tilde{p}(\tilde{r},\tilde{t})}{\sqrt{1-\tilde{r}^2}} \, d\tilde{r} . \end{aligned}$$

Note that through proper manipulation of (24) and the use of (25), (22)$_2$ can be expressed in the following form:

26 $$\begin{aligned} \tilde{w}(\tilde{r},\tilde{t}) = \frac{8}{\pi }L(\tilde{t}) \int _0^1 \frac{\partial \tilde{p}}{\partial y}(y,\tilde{t}) {{\mathcal {K}}}(y,\tilde{r}) \, dy + \frac{4}{\sqrt{\pi }}\sqrt{L(\tilde{t})}\tilde{K}_I \sqrt{1-\tilde{r}^2} , \end{aligned}$$

for the kernel function ${\mathcal {K}}$ given by:

27 $${\mathcal{K}}(y,\tilde{r}) = y\left[ {E\left( {\arcsin \left( y \right)\left| {\frac{{\tilde{r}^{2} }}{{y^{2} }}} \right.} \right) - E\left( {\arcsin \left( \chi \right)\left| {\frac{{\tilde{r}^{2} }}{{y^{2} }}} \right.} \right)} \right],$$

where:

28 $$\begin{aligned} \chi = \min \left( 1,\frac{y}{\tilde{r}}\right) , \end{aligned}$$

with the function $E\left( {\phi \left| m \right.} \right)$ denoting the incomplete elliptic integral of the second kind [29].

While this form of the elasticity operator has not previously been used in the case of a penny-shaped fracture, an analogous form of the elasticity equation for the KGD model has been utilized in [24, 25], where its advantages in numerical computations have been demonstrated. Notably, the kernel function ${\mathcal {K}}$ exhibits better behaviour than the weakly singular kernel *G* (7), having no singularities for any combination of $\left\{ \tilde{r},y\right\}$. Additionally, Eq. (19) can be easily transformed to obtain $p^\prime$ and then substituted into (26), meaning that the latter can be utilized without the additional step of deriving the pressure function needed for the classic form of the operator.

Next the boundary conditions (9), in view of (15), transform to a single condition:

29 $$\begin{aligned} \tilde{w}(1,\tilde{t})=0, \end{aligned}$$

alongside the initial conditions (10):

30 $$\begin{aligned} \tilde{w}(\tilde{r},0) = \frac{w_*(r)}{l_*} , \quad L_0=\frac{l_0}{l_*} . \end{aligned}$$

The source strength (8) is now defined as:

31 $$\begin{aligned} \frac{\tilde{Q}_0(\tilde{t})}{2\pi }=\lim _{\tilde{r}\rightarrow 0}\tilde{r}\tilde{w}(\tilde{r},\tilde{t})\tilde{v}(\tilde{r},\tilde{t}) . \end{aligned}$$

While combining the above with (19) we obtain the following relationship:

32 $$\begin{aligned} \lim _{\tilde{r}\rightarrow 0}\tilde{r}^n \frac{\partial \tilde{p}}{\partial \tilde{r}} =- \left( \frac{\tilde{Q}_0 (\tilde{t})}{2\pi } \right) ^{n} \frac{L (\tilde{t})}{\tilde{w}^{2n+1} (0,\tilde{t})} , \end{aligned}$$

which provides a valuable insight into how the behaviour of the fluid pressure function near to the source varies for differing values of *n*. The resulting pressure asymptotics at the injection point, with corresponding aperture, are detailed below:

33 $$\begin{aligned} \tilde{p}(\tilde{r},\tilde{t})= & {} \tilde{p}_0^o (\tilde{t}) + \tilde{p}_1^o (\tilde{t}) \tilde{r}^{1-n} + O\left( \tilde{r}^{2-n} \right) , \quad \tilde{r}\rightarrow 0 , \end{aligned}$$

34 $$\begin{aligned} \tilde{w}(\tilde{r},\tilde{t})= & {} \tilde{w}_0^o (\tilde{t}) +\tilde{w}_1^o (\tilde{t}) \tilde{r}^{2-n} + O\left( \tilde{r}^2 \log (\tilde{r})\right) , \quad \tilde{r}\rightarrow 0 . \end{aligned}$$

It is worth restating that there are minor differences to both the asymptotics and fundamental equations in the limiting cases $n=0$ and $n=1$. These are explained in further detail in “Appendix 1”.

## Crack tip asymptotics, the speed equation and proper variables

A universal algorithm for numerically simulating hydraulic fractures has recently been introduced in [24, 25] and tested against the PKN and KGD (plane strain) models for Newtonian and shear-thinning fluids. It proved to be extremely efficient and accurate. Its modular architecture enables one to adapt it to other HF models by simple replacement or adjustment of the basic blocks. In the following we will construct a computational scheme for the radial fracture based on the universal algorithm. To this end we need to introduce appropriate computational variables, and to define the basic asymptotic interrelations between them. For the sake of completeness detailed information on the solutions tip asymptotic behaviour, for different regimes of crack propagation, are presented below.

### Crack tip asymptotics

#### Viscosity dominated regime ($\tilde{K}_{Ic} = 0$)

In the viscosity dominated regime the crack tip asymptotics of the aperture and pressure derivative can be expressed as follows:

35 $$\begin{aligned} \tilde{w}(\tilde{r},\tilde{t})&= \tilde{w}_0(\tilde{t}) \left( 1-\tilde{r}^2 \right) ^{\alpha _0} +\, \tilde{w}_1(\tilde{t})\left( 1-\tilde{r}^2 \right) ^{\alpha _1}+\, \tilde{w}_2(\tilde{t})\left( 1-\tilde{r}^2 \right) ^{\alpha _2} \nonumber \\&\quad+\, O\left( \left( 1-\tilde{r}^2 \right) ^{\alpha _2 + \delta }\right) , \quad \tilde{r} \rightarrow 1, \end{aligned}$$

36 $$\begin{aligned} \frac{\partial \tilde{p}}{\partial \tilde{r}} (\tilde{r}, \tilde{t})= & {} \,\tilde{p}_0 (\tilde{t}) \left( 1-\tilde{r}^2\right) ^{\alpha _0-2} +\, \tilde{p}_1 (\tilde{t}) \left( 1-\tilde{r}^2\right) ^{\alpha _0-1} +\, O\left( 1 \right) , \quad \tilde{r}\rightarrow 1 . \end{aligned}$$

The crack tip asymptotics of the pressure function can be derived from the above, however this form is given due to its use in computations (this will be explained in further detail later).

As a consequence the fluid velocity behaves as:

37 $$\begin{aligned} \tilde{v} (\tilde{r} , \tilde{t} ) = \tilde{v}_0 (\tilde{t} ) + \tilde{v}_1 \left( \tilde{t} \right) \left( 1-\tilde{r}^2 \right) ^{\beta _1} + O\left( \left( 1-\tilde{r}^2 \right) ^{\beta _2}\right) , \quad \tilde{r} \rightarrow 1 . \end{aligned}$$

Note that we require $\tilde{v}_0 (\tilde{t} )>0$ to ensure the fracture is moving forward. The values of constants $\alpha _i$, $\beta _i$ are given in Table 1. The general formulae for the limiting cases $n=0$ and $n=1$ remain the same as (35)-(37), with the respective powers $\alpha _i$, $\beta _i$ again being determined according to Table 1.

Now, let us adopt the following notation for the crack propagation speed, based on the *speed equation* (20) and the tip asymptotics (37):

38 $$\begin{aligned} \tilde{v}_0 (\tilde{t}) = L^\prime (\tilde{t}) = \left[ \frac{{\mathcal {C}} {\mathcal {L}}(\tilde{w} )}{L^2 ( \tilde{t} )} \right] ^{\frac{1}{n}} . \end{aligned}$$

Here ${\mathcal{{L}}}(\tilde{w})>0$ is a known functional and $\mathcal{{C}}$ is a positive constant. In the viscosity dominated regime we have that:

39 $$\begin{aligned} C = \frac{2n}{(n+2)^2} \cot \left( \frac{n\pi }{n+2}\right) , \quad {\mathcal{{L}}} (\tilde{w}) = \tilde{w}_0^{n+2} . \end{aligned}$$

Additionally, we can directly integrate (38) in order to obtain an expression for the fracture length:

40 $$\begin{aligned} L(\tilde{t}) = \left[ L^{1+\frac{2}{n}}(0) + \left( 1+\frac{2}{n}\right) {\mathcal {C}}^{\frac{1}{n}} \int _0^{\tilde{t}} {\mathcal {L}}^{\frac{1}{n}} (\tilde{w}) \, d\tau \right] ^{\frac{n}{n+2}} . \end{aligned}$$

**Table 1:** Values of the basic constants used in the asymptotic expansions for $\tilde{w}$ and $\tilde{v}$ for $0<n<1$

Crack propagation regime	$\alpha _0$	$\alpha _1$	$\alpha _2$	$\beta _1$	$\beta _2$
Viscosity dominated	$\dfrac{2}{n+2}$	$\dfrac{n+4}{n+2}$	$\dfrac{2n+6}{n+2}$	1	$\dfrac{2n+2}{n+2}$
Toughness dominated	$\dfrac{1}{2}$	$\dfrac{3-n}{2}$	$\dfrac{5-2n}{2}$	$\dfrac{2-n}{2}$	1

#### Toughness dominated regime ($\tilde{K}_{Ic} > 0$)

Near the fracture front the forms of the aperture and fluid velocity asymptotics remain the same as in the viscosity dominated regime (35), (37). Meanwhile the pressure derivative asymptotics yields:

41 $$\begin{aligned} \frac{\partial \tilde{p}}{\partial \tilde{r}} (\tilde{r}, \tilde{t}) = \tilde{p}_0 \left( 1-\tilde{r}^2\right) ^{\alpha _1-2} +\, \tilde{p}_1 \left( 1-\tilde{r}^2\right) ^{\alpha _2-2} +\, O\left( 1 \right) , \quad \tilde{r}\rightarrow 1 . \end{aligned}$$

The values of $\alpha _i$, $\beta _i$ for this regime are provided in Table 1. The limiting cases $n=0$ and $n=1$ are discussed in “Appendix 1” (Eqs. 78, 87 respectively).

We again use notation (38) for the crack propagation speed, however the values of the functional ${\mathcal {L}}$ and the *C* will in this case be:

42 $$\begin{aligned} C= \frac{(3-n)(1-n)}{4} \tan \left( \frac{n\pi }{2}\right) , \quad {\mathcal {{L}}}(\tilde{w}) = \tilde{w}_0^{n+1} \tilde{w}_1 , \end{aligned}$$

while the fracture length will be given by (40).

### Reformulation in terms of computational variables

It is readily apparent that the choice of computational variables plays a decisive role in ensuring the accuracy and efficiency of the computational algorithm [23, 24, 26]. Let us introduce a new system of proper variables which are conducive to robust numerical computing.

- The reduced fluid velocity $\Phi (\tilde{r},\tilde{t})$:
  43 $$\begin{aligned} \Phi (\tilde{r},\tilde{t})=\tilde{r}\tilde{v}(\tilde{r},\tilde{t})-\tilde{r}^{2} \tilde{v}_0(\tilde{t}) . \end{aligned}$$
  It is a smooth, well behaved and non-singular variable that facilitates the numerical computations immensely. It is bounded at the crack tip and the fracture origin. The advantages of using an analogous variable in the PKN and KGD models have previously been demonstrated in [24, 25].
- The modified fluid pressure derivative $\Omega (\tilde{r},\tilde{t})$:
  44 $$\begin{aligned} \tilde{r}^n \Omega (\tilde{r},\tilde{t})= & {} \tilde{r}^n \frac{\partial \tilde{p}}{\partial \tilde{r}}-\Omega _0(\tilde{t}), \end{aligned}$$
  45 $$\begin{aligned} \Omega _0 (\tilde{t})= & {} - \left( \frac{\tilde{Q}_0 (\tilde{t})}{2\pi } \right) ^{n} \frac{L (\tilde{t})}{\tilde{w}^{2n+1} (0,\tilde{t})}. \end{aligned}$$
  It reflects the singular tip behavior of $\tilde{p}'_{\tilde{r}}$, having the same tip asymptotics as the pressure derivative, however it is bounded at the fracture origin. From (44) the pressure can be immediately reconstructed as:
  46 $$\begin{aligned} \tilde{p}(\tilde{r},\tilde{t})= \frac{\Omega _0(\tilde{t})}{1-n} \tilde{r}^{1-n}+C_p(\tilde{t})+\int _0^{\tilde{r}}\Omega (\xi , \tilde{t} )d\xi , \end{aligned}$$
  where the term $C_p$ follows from (25):
  47 $$\begin{aligned} C_p (\tilde{t}) = \frac{1}{2}\sqrt{\frac{\pi }{L(\tilde{t})}} \tilde{K}_I - \frac{\sqrt{\pi }\Gamma \left( \frac{3-n}{2}\right) }{2\left( 1-n\right) \Gamma \left( 2-\frac{n}{2}\right) } \Omega _0 (\tilde{t}) - \int _0^1 \Omega (y , \tilde{t})\sqrt{1-y^2} \, dy . \end{aligned}$$
  This auxiliary variable will primarily be used in numerical computation of the elasticity operator.

The following interrelationship exists between the newly introduced variables:

48 $$\begin{aligned} \Omega (\tilde{r},\tilde{t}) = \left( \frac{\tilde{Q}_0 (\tilde{t})}{2\pi \tilde{r}} \right) ^{n} \frac{L(\tilde{t})}{\tilde{w}^{2n+1} (0,\tilde{t})} - \frac{L(\tilde{t})}{\tilde{w}^{n+1}(\tilde{r},\tilde{t})}\left[ \frac{\Phi (\tilde{r},\tilde{t})}{\tilde{r}} + \tilde{r} \tilde{v}_0(\tilde{t}) \right] ^{n} . \end{aligned}$$

Since under this new scheme $\Phi$ is bounded at the fracture tip, the source strength (31) and the boundary condition (29) can now be expressed as:

49 $$\begin{aligned} \tilde{w} (0,\tilde{t}) \Phi (0 , \tilde{t} ) = \frac{\tilde{Q}_0 (\tilde{t})}{2\pi } , \quad \tilde{w} (1, \tilde{t} ) = 0 . \end{aligned}$$

By utilizing the boundary condition (49)$_1$, the relationship between the new variables (48) can be represented in the form:

50 $$\begin{aligned} \Omega \left( \tilde{r},\tilde{t}\right) = \frac{1}{\tilde{r}^n} \left[ \frac{ \Phi ^n (0,\tilde{t})}{\tilde{w}^{n+1} (0,\tilde{t})} - \frac{\left( \Phi (\tilde{r},\tilde{t})+\tilde{r}^2 \tilde{v}_0 (\tilde{t}) \right) ^n }{\tilde{w}^{n+1} (\tilde{r},\tilde{t})} \right] . \end{aligned}$$

Note that this is not only a more concise representation of (48) but it also does not depend on $L(\tilde{t})$, which will be beneficial when analyzing the self-similar formulation. In this way the computational scheme will be based on: the crack opening, $\tilde{w}$, the reduced fluid velocity, $\Phi$, and an auxiliary variable, the modified fluid pressure, $\Omega$.

By substituting the new variable $\Phi$ from (43) into the continuity Eq. (18), we obtain the modified governing equation:

51 $$\begin{aligned} \frac{\partial \tilde{w}}{\partial \tilde{t}} + \frac{1}{L(\tilde{t})\tilde{r}} \frac{\partial }{\partial \tilde{r}}\left( \tilde{w} \Phi \right) + \frac{2 \tilde{v}_0}{L(\tilde{t})} \tilde{w} + \tilde{q}_l = 0 , \quad 0<\tilde{r}<1 . \end{aligned}$$

Additionally, the elasticity Eq. (26) can be now expressed as follows:

52 $$\begin{aligned} \tilde{w}(\tilde{r},\tilde{t}) = \frac{8}{\pi }L(\tilde{t}) \int _0^1 \Omega (y,\tilde{t}) {\mathcal {K}}(y,\tilde{r}) \, dy +\frac{4}{\sqrt{\pi }}\sqrt{L(\tilde{t})} \tilde{K}_I \sqrt{1-\tilde{r}^2} + \frac{8}{\pi }L(\tilde{t}) \Omega _0 (\tilde{t}) {\mathcal {G}}_n (\tilde{r}) , \end{aligned}$$

where ${\mathcal {K}}$ is given in (27), while $\mathcal {G}_n$ is defined by:

53 $$\begin{aligned} {\mathcal {G}}_n (\tilde{r}) = \frac{\sqrt{\pi } \Gamma \left( \frac{3-n}{2}\right) }{2\left( n-1\right) \Gamma \left( 2-\frac{n}{2}\right) }\left[ \sqrt{1-\tilde{r}^2} + \frac{{_2F_1}\left( \frac{1}{2},\frac{n-2}{2};\frac{n}{2};\tilde{r}^2\right) }{n-2} - \frac{\sqrt{\pi }\tilde{r}^{2-n} \Gamma \left( \frac{n}{2}-1\right) }{2\Gamma \left( \frac{n-1}{2}\right) }\right] . \end{aligned}$$

It can be easily shown that this function is well behaved in the limits.

## Self-similar formulation

In this section we will reduce the problem to its time-independent self-similar version. For this formulation we will define the computational scheme used later on in the numerical analysis.

We begin by assuming that a solution to the problem can be expressed in the following manner:

54 $$\begin{aligned} \tilde{w}(\tilde{r},\tilde{t})= & {} \Psi (\tilde{t})\hat{w}(\tilde{r}) ,\quad \tilde{p}(\tilde{r},\tilde{t})=\frac{\Psi (\tilde{t})}{L(\tilde{t})}\hat{p} (\tilde{r}), \quad \tilde{q}(\tilde{r},\tilde{t})=\frac{\Psi ^{2+\frac{2}{n}} (\tilde{t})}{L^{\frac{2}{n}}(\tilde{t})}\hat{q}(\tilde{r}), \nonumber \\ \tilde{Q}_0 (\tilde{t})= & {} \frac{\Psi ^{2+\frac{2}{n}} (\tilde{t})}{L^{\frac{2}{n}}(\tilde{t})} \hat{Q}_0, \quad \tilde{v}(\tilde{r},\tilde{t})=\frac{\Psi ^{1+\frac{2}{n}}(\tilde{t})}{L^{\frac{2}{n}}(\tilde{t})}\hat{v}(\tilde{r}), \quad \Phi (\tilde{r}, \tilde{t})=\frac{\Psi ^{1+\frac{2}{n}}(\tilde{t})}{L^{\frac{2}{n}}} \hat{\Phi }(\tilde{r}), \nonumber \\ \tilde{K}_I(\tilde{t})= & {} \frac{\Psi (\tilde{t})}{\sqrt{L(\tilde{t})}} \hat{K}_I, \quad \Omega (\tilde{r},\tilde{t}) = \frac{\Psi (\tilde{t})}{L(\tilde{t})} \hat{\Omega }(\tilde{r}) ,\nonumber \\ \Omega _0 (\tilde{t})= & {} \frac{\Psi (\tilde{t})}{L(\tilde{t})} \hat{\Omega }_0 , \quad C_p (\tilde{t}) = \frac{\Psi (\tilde{t})}{L(\tilde{t})} \hat{C}_p , \end{aligned}$$

where $\Psi (t)$ is a smooth continuous function of time. Such a separation of variables enables one to reduce the problem to a time-independent formulation when $\Psi$ is described by an exponential or a power-law type function. From here on the spatial components will be marked by a ’hat’-symbol, and will describe the self-similar quantities. It is worth noting that the separation of spatial and temporal components given in (54) ensures that the qualitative behaviour of the solution tip asymptotics remains the same as in the time-dependent variant.

### The self-similar representation of the problem

We wish to examine two variants of the time dependent function:

55 $$\begin{aligned} \Psi _1 (\tilde{t}) = e^{\gamma \tilde{t}} , \quad \Psi _2 (\tilde{t} ) = \left( a + \tilde{t} \right) ^{\gamma } . \end{aligned}$$

In both cases the fluid leak-off function will be assumed to take the form:

56 $$\begin{aligned} \tilde{q}_l (\tilde{r} , \tilde{t} ) = \frac{1}{\gamma } \Psi ^\prime (\tilde{t} ) \hat{q}_l (\tilde{r} ) . \end{aligned}$$

The self-similar reduced fluid velocity (43), modified fluid pressure derivative (44), (45) and pressure (46) are defined by:

57 $$\begin{aligned} \hat{\Phi }(\tilde{r})= & {}\, \tilde{r} \hat{v}(\tilde{r}) - \tilde{r}^2 \hat{v}_0 , \quad \tilde{r}\hat{\Omega }(\tilde{r}) = \tilde{r}\frac{d\hat{p}}{d\tilde{r}}-\hat{\Omega }_0 , \end{aligned}$$

58 $$\begin{aligned} \hat{p}(\tilde{r})= & {} \frac{\hat{\Omega }_0}{1-n} \tilde{r}^{1-n}+\hat{C}_p+\int _0^{\tilde{r}} \hat{\Omega }(\xi )d\xi , \end{aligned}$$

with

59 $$\begin{aligned} \hat{\Omega }_0= & {} - \left( \frac{\hat{Q}_0}{2\pi } \right) ^{n} \frac{1}{\hat{w}^{2n+1} (0)} , \end{aligned}$$

60 $$\begin{aligned} \hat{C}_p= & {} \frac{\sqrt{\pi }}{2}\hat{K}_I - \frac{\sqrt{\pi }\Gamma \left( \frac{3-n}{2}\right) }{2\left( 1-n\right) \Gamma \left( 2-\frac{n}{2}\right) } \hat{\Omega }_0 - \int _0^1 \hat{\Omega } (y)\sqrt{1-y^2} \, dy . \end{aligned}$$

It is immediately apparent from (38) and (54) that the self-similar crack propagation speed is given by:

61 $$\begin{aligned} \hat{v}_0 = \lim _{\tilde{r}\rightarrow 1} \left[ - \hat{w}^{n+1} \frac{d \hat{p}}{d \tilde{r}}\right] ^{\frac{1}{n}} = \left( {\mathcal {C}}\mathcal {L}(\hat{w}) \right) ^{\frac{1}{n}} . \end{aligned}$$

Note again that the qualitative asymptotic behaviour of the aperture, pressure and fluid velocity as $\tilde{r}\rightarrow 0$ and $\tilde{r}\rightarrow 1$ remains the same as in the time dependent version of the problem (35), (36), (37), (41). In the self-similar formulation, the multipliers of respective terms are time-independent.

The self-similar counterparts of the elasticity Eqs. (22) and (23) are now:

62 $$\begin{aligned} \hat{p} ( \tilde{r} ) = \hat{\mathcal {A}} [\hat{w} ] (\tilde{r} ) , \end{aligned}$$

where

63 $$\begin{aligned} \hat{\mathcal {A}} [\hat{w} ] ( \tilde{r} ) = - \int _0^1 \frac{d \hat{w} ( \eta )}{d \eta } M \left[ \tilde{r} , \eta \right] \, d\eta , \end{aligned}$$

with the inverse relation being:

64 $$\begin{aligned} \hat{w}(\tilde{r}) = \frac{8}{\pi } \int _0^1 \hat{\Omega }(y) {\mathcal {K}}(y,\tilde{r}) \, dy + \frac{4}{\sqrt{\pi }} \hat{K}_I \sqrt{1-\tilde{r}^2} + \frac{8}{\pi } \hat{\Omega }_0 {\mathcal {G}}_n (\tilde{r}). \end{aligned}$$

As the integral and function ${\mathcal {G}}_n (\tilde{r})$ both tend to zero faster than the square root term at the fracture tip, it immediately follows that, in the toughness dominated case ($\hat{K}_{Ic}>0$), the leading asymptotic term of the aperture (35) is given by:

65 $$\begin{aligned} \hat{w}_0 = \frac{4}{\sqrt{\pi }}\hat{K}_I . \end{aligned}$$

The self-similar fluid velocity takes the form:

66 $$\begin{aligned} \hat{v}(\tilde{r}) = \left[ - \hat{w}^{n+1} (\tilde{r}) \frac{d \hat{p}(\tilde{r})}{d \tilde{r}} \right] ^{\frac{1}{n}} . \end{aligned}$$

The governing Eq. (51) becomes:

67 $$\begin{aligned} \frac{1}{\tilde{r} \hat{v}_0} \frac{d}{d\tilde{r}} \left( \hat{w} \hat{\Phi } \right) = -\left( 3 - \rho \right) \hat{w} - \left( 1-\rho \right) \frac{\hat{q}_l}{\gamma }, \end{aligned}$$

with the value of $\rho$ in each case, alongside the fracture length, provided in Table 2. Meanwhile the fluid balance condition (21) becomes:

68 $$\begin{aligned} \left( 3-\rho \right) \int _0^1 \tilde{r} \hat{w} ( \tilde{r} ) \, d \tilde{r} + \frac{1-\rho }{\gamma } \int _0^1 \tilde{r} \hat{q}_l \ d\tilde{r} = \frac{\hat{Q}_0}{2\pi \hat{v}_0} . \end{aligned}$$

**Table 2:** Table providing the fracture length $L(\tilde{t})$, which is obtained using (40) and (61), and the constant $\rho$, used in (67) and (68), for different variants of the self-similar solution

Self-similar law	$\rho$	$L(\tilde{t})$
$\Psi (\tilde{t} ) = e^{\gamma \tilde{t}}$	0	$\left[ \frac{\hat{v}_0}{\gamma } \right] ^{\frac{n}{n+2}} e^{\gamma \tilde{t} }$
$\Psi (\tilde{t} ) = \left( a + \tilde{t} \right) ^{\gamma }$	$\frac{n}{\gamma \left( n+2\right) +n}$	$\left[ \frac{\left( n+2\right) \hat{v}_0}{\gamma \left( n+2\right) +n}\right] ^{\frac{n}{n+2}} \left( a+\tilde{t}\right) ^{\gamma +\frac{n}{n+2}}$

The self-similar stress intensity factor (25) is given by:

69 $$\begin{aligned} \hat{K}_I = \hat{K}_{Ic} = \, \frac{2}{\sqrt{\pi }} \int _0^1 \frac{\tilde{r}\hat{p}(\tilde{r})}{\sqrt{1-\tilde{r}^2}} \, d\tilde{r} . \end{aligned}$$

Finally, the system’s boundary conditions (49) transform to:

70 $$\begin{aligned} \hat{w} (0) \hat{\Phi } (0 ) = \frac{\hat{Q}_0 }{2\pi } , \quad \hat{w} (1 ) = 0 . \end{aligned}$$

In the general case with $0<n<1$ these equations represent the full self-similar problem. Some modifications are necessary in the special cases when $n=0$ and $n=1$. These differences are outlined in “Appendix 1”.

## Numerical results

In this section we will construct an iterative computational scheme for numerically simulating hydraulic fracture. The approach is an extension of the universal algorithm introduced in [24, 25]. The computations are divided between two basic blocks, the first of which utilizes the continuity equation and the latter using the elasticity operator. The previously introduced computational variables, alongside the known information about the solution tip asymptotics, are employed extensively. The accuracy and efficiency of the computations are verified against the newly introduced analytical benchmark examples. Then the numerical benchmark solutions are given. Finally, a comparative analysis with other data available in the literature is delivered.

### Computational scheme

The algorithm is constructed using the approach framework introduced for the PKN and KGD models in [24, 25]. The numerical scheme is realized as follows:

1. An initial approximation of the aperture $\hat{w}=\hat{w}^{j-1}$ is taken, such that it has the correct asymptotic behaviour and satisfies the boundary conditions.
2. The fluid balance Eq. (68) is utilized to obtain the asymptotic term(s) $\hat{w}_{0,1}^{j}$ needed to compute the fluid velocity $\hat{v}_0^{j}$ using (61).
3. Having the above values the reduced fluid velocity $\hat{\Phi }^{j}$ is reconstructed by direct integration of (67). Tikhonov type regularization is employed at this stage.
4. Equations (57) and (66) is then used to obtain an approximation of the modified fluid pressure derivative $\hat{\Omega }$, and the elasticity Eq. (64) serves to compute the next approximation of the fracture aperture $\hat{w}^j$.
5. The system is iterated until all variables $\hat{\Phi }$, $\hat{w}$ and $\hat{v}_{0}$ converge to within prescribed tolerances.

We will demonstrate in this section that this scheme, combined with an appropriate meshing strategy, yields a highly accurate algorithm. A more detailed description of the algorithm’s construction has been outlined in [24, 25]. When iterated, the system of discretized equations converges to a final solution in under 20 iterations, with computation times on a standard laptop under 5 s when taking $N=20$, and under 30 s when $N=300$.

It is worth noting that, due to the degeneration of the Poiseuille equation when $n=0$, it can no longer be used to compute the fluid flow rate or the fluid velocity. However, thanks to the modular structure of the proposed algorithm, one can easily adapt it to this variant of the problem. In this case a special form of the elasticity Eq. (96) is utilized to obtain the aperture, with the fluid velocity being reconstructed using relations (97) and (98).

### Accuracy of computations

In this subsection we will investigate the accuracy of computations delivered by the proposed numerical scheme. To this end a newly introduced set of analytical benchmark solutions with a non-zero fluid leak-off function will be used. Alternative measures for testing the numerical accuracy in the absence of exact solutions will then be proposed and analysed. Next, the problem of a penny-shaped hydraulic fracture propagating in an impermeable material will be considered. The accuracy of numerical solutions will be verified by the aforementioned alternative measures. Simple, semi-analytical approximations, which mimic the obtained numerical data to a prescribed level of accuracy will be provided in the second part of this paper, alongside a comparative analysis with other solutions available in the literature.

#### Analysis of computational errors against analytical benchmarks

The first method of testing the computational accuracy is by comparison with analytical benchmark solutions. Respective closed-form benchmarks with predefined, non-zero, leak-off functions are outlined in the supplementary material associated with this paper. They have been constructed for both the viscosity and toughness dominated regimes, for a class of shear-thinning and Newtonian fluids. All of the analytical benchmarks used for comparison are designed to ensure physically realistic behaviour of the solution while maintaining the proper asymptotic behaviour. In all numerical simulations the power-law form of the time dependent function $\Psi _2$ (55)$_2$ is used to ensure consistency with results in the literature (e.g. [19]).

**Fig. 1:**
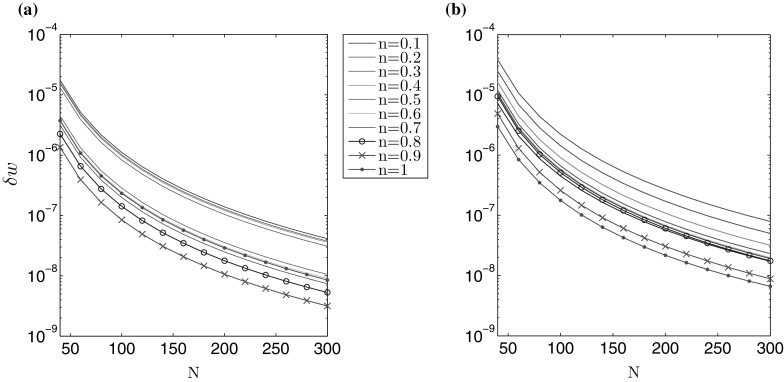
Relative average error of the crack aperture (71)$_1$ obtained against the analytical benchmark over *N* for the **a** viscosity dominated regime, **b** toughness dominated regime

**Fig. 2:**
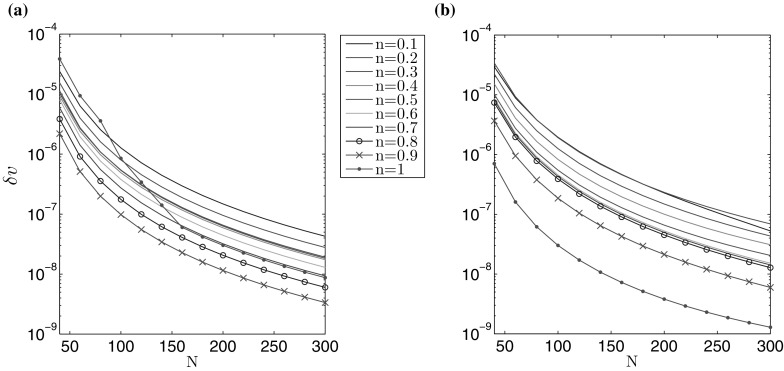
Relative average error of the fluid velocity (71)$_2$ obtained against the analytical benchmark over *N* for the **a** viscosity dominated regime, **b** toughness dominated regime

The accuracy of computations is depicted in Figs. 1 and 2, for varying number of nodal points *N*. A non-uniform spatial mesh was used, with meshing density increased near the ends of the interval (the same type of mesh was used for all *n*). The measures $\delta w$, $\delta v$, describing the average relative error of the crack opening and fluid velocity, are taken to be:

71 $$\begin{aligned} \delta w (N) = \frac{\int _0^1 \tilde{r} \left| \hat{w}^* (\tilde{r}) - \hat{w}(\tilde{r}) \right| \, d\tilde{r} }{\int _0^1 \tilde{r} \hat{w}^* (\tilde{r}) \, d\tilde{r}} , \quad \delta v (N) = \frac{\int _0^1 \tilde{r} \left| \hat{v}^* (\tilde{r}) - \hat{v}(\tilde{r}) \right| \, d\tilde{r} }{\int _0^1 \tilde{r} \hat{v}^* (\tilde{r}) , d\tilde{r}} , \end{aligned}$$

where $\hat{w}^*$ and $\hat{v}^*$ denote the exact solutions for $\hat{w}$ and $\hat{v}$.

The results clearly show that the values of both error measures decrease monotonically with growing *N*. For a fixed number of nodal points *N*, $\delta w$ is lower than $\delta v$, but within the same order of magnitude. One can observe a sensitivity of the results to the value of the fluid behaviour index *n*. Here, the level of error measures can vary up to one order for a constant *N*. This trend can be alleviated by adjusting the mesh density distribution to the value of *n* (i.e. to the varying asymptotics of solution), however such an investigation goes beyond the scope of this paper. In general, it takes fewer than $N=300$ nodal points to achieve the relative errors of the level $10^{-7}$.

In cases when the exact solution is not prescribed an alternative method of testing the solution accuracy is required. The method outlined here relies on the fact that the solution converges to the exact value at a known rate, with respect to the number of nodal points. The convergence rate has been established numerically to behave as $1/N^3$. As a result the following estimation holds:

72 $$\begin{aligned} \int _0^1 \tilde{r} \hat{g}_i(\tilde{r}) \, d\tilde{r}=A_i+\frac{B_i}{N^3}, \quad i=1,2 , \end{aligned}$$

where $\hat{g}_1(\tilde{r})=\hat{w}(\tilde{r})$ and $\hat{g}_2(\tilde{r})=\hat{v}(\tilde{r})$. $A_i$ and $B_i$ are constants which can be found numerically through use of a least-squares approximation. Next, one can define the limiting value of (72) as:

73 $$\begin{aligned} \lim _{N \rightarrow \infty } \int _0^1 \tilde{r} \hat{g}_i(\tilde{r}) \, d\tilde{r}=A_i \approx \int _0^1 \tilde{r} \hat{g}^*_i(\tilde{r}) \, d\tilde{r}, \quad i=1,2 , \end{aligned}$$

for $$\hat{g}^*_1(\tilde{r})=\hat{w}^*(\tilde{r})$$, $$\hat{g}^*_2(\tilde{r})=\hat{v}^*(\tilde{r})$$.

Knowing this, the following alternative error measures can be proposed:

74 $$\begin{aligned} e_{{g}_i}(N) = \left| 1 -\frac{1}{A_i} \int _0^1 \tilde{r} \hat{g}_i (\tilde{r}) \, d\tilde{r} \right| ,\quad i=1,2. \end{aligned}$$

**Fig. 3:**
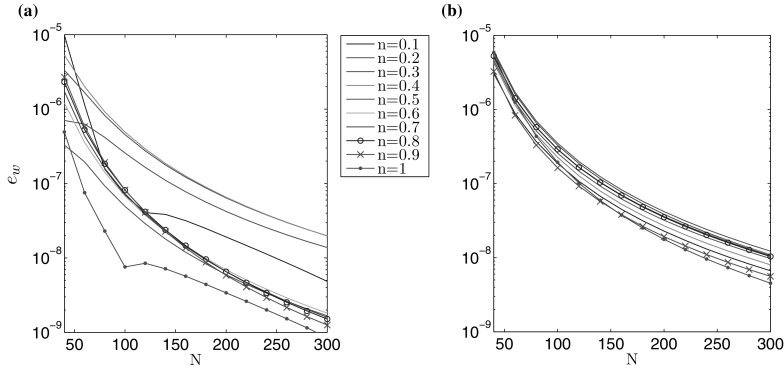
Rate of convergence $e_w$ (74) of the numerical solution for the benchmark example: **a** viscosity dominated regime, **b** toughness dominated regime

**Fig. 4:**
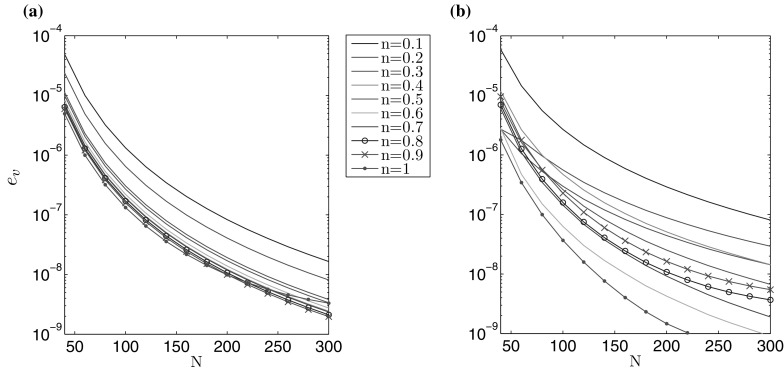
Rate of convergence $e_v$ (74) of the numerical solution for the benchmark example: **a** viscosity dominated regime, **b** toughness dominated regime

Using this strategy, it is possible to identify the relative rate at which the solution converges: $e_w (N)$ for the aperture and $e_v (N)$ for the fluid velocity. The results are shown in Figs. 3 and 4. It is notable that both $\delta w$ and $e_w$, as well as $\delta v$ and $e_v$, provide estimates of a similar order for a fixed N. Thus, both of these error measures can be employed in the accuracy analysis, although only the latter ($e_{w,v}$) in the cases when no exact solution is available. As such, $e_w (N)$ and $e_v (N)$ will be used in the following investigations (it should also be noted that this approach has previously been shown to be successful for the PKN/KGD models of HF [24, 25, 33]).

#### Impermeable solid: reference solutions

With a suitable measure for testing the solution accuracy in place we move onto examining the solution variant most frequently studied in the literature, the case with a zero valued leak-off function and with $\hat{Q}_0=1$. Although there is no analytical solution to this problem, due to its relative simplicity, it is commonly used when testing numerical algorithms. For this reason it is very important that credible reference data is provided for this case, which can be easily employed to verify various computational schemes. Both the viscosity and toughness dominated regimes (for different values of the material toughness: $\hat{K}_{Ic}=\left\{ 1,10,100\right\}$) will be investigated. In the second part of the paper, accurate and simple approximations of the obtained numerical solutions will be provided.

**Fig. 5:**
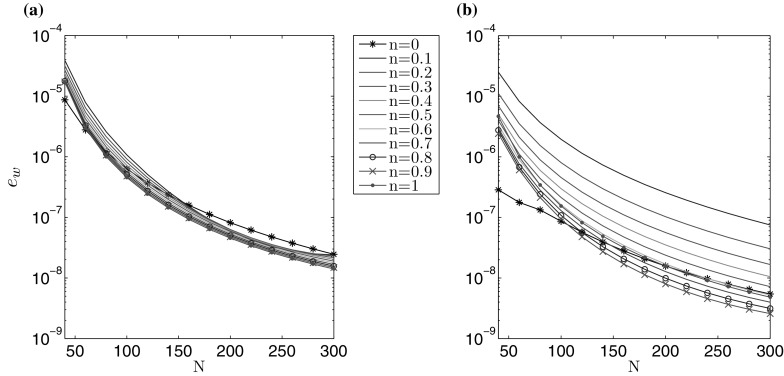
Rate of convergence $e_w$ (74) of the numerical solution when $Q_0=1$ with no fluid leak-off for the: **a** viscosity dominated regime, **b** toughness dominated regime with $\hat{K}_{Ic}=1$

**Fig. 6:**
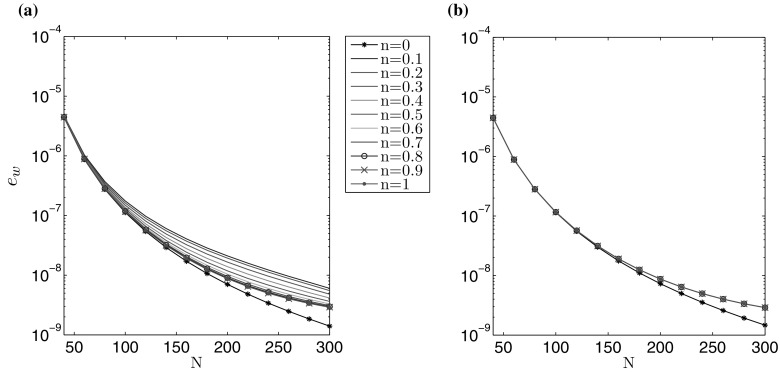
Rate of convergence $e_w$ (74) of the numerical solution when $Q_0=1$ with no fluid leak-off for the toughness dominated regime with: **a** $\hat{K}_{Ic}=10$ and **b** $\hat{K}_{Ic}=100$

**Fig. 7:**
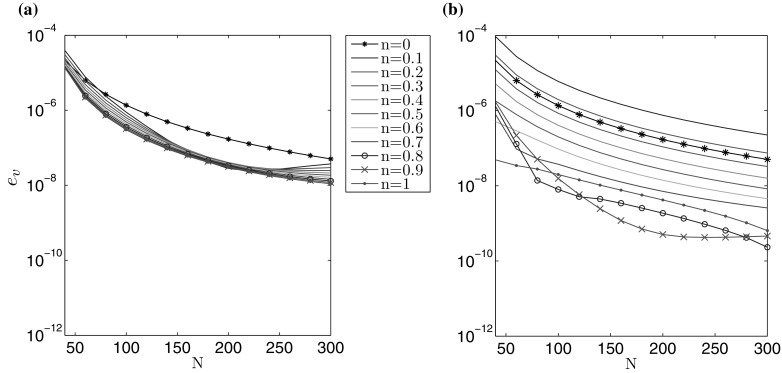
Rate of convergence $e_v$ (74) of the numerical solution when $Q_0=1$ with no fluid leak-off for the: **a** viscosity dominated regime, **b** toughness dominated regime with $\hat{K}_{Ic}=1$

**Fig. 8:**
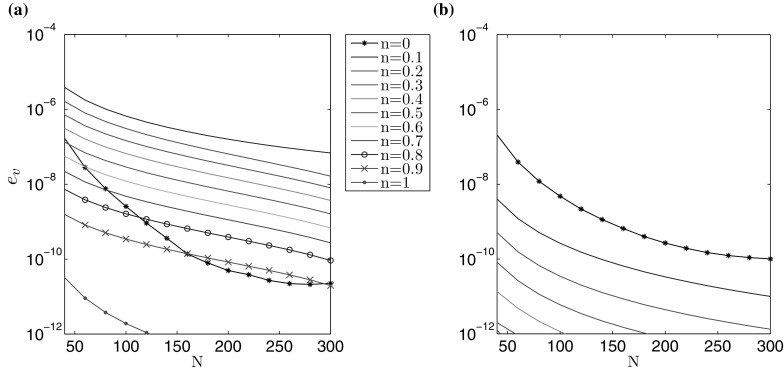
Rate of convergence $e_v$ (74) of the numerical solution when $Q_0=1$ with no fluid leak-off for the toughness dominated regime with: **a** $\hat{K}_{Ic}=10$ and **b** $\hat{K}_{Ic}=100$

The results for the crack opening and fluid velocity convergence rates are shown in Figs. 5, 6, 7 and 8.

**Fig. 9:**
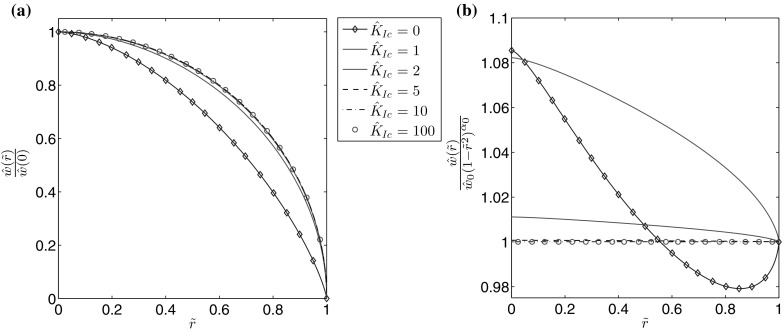
The aperture for $n=0.5$ for a different values of the fracture toughness: **a** the normalized self-similar aperture, **b** the self-similar aperture divided by the leading term of the crack tip asymptotics (35)

**Fig. 10:**
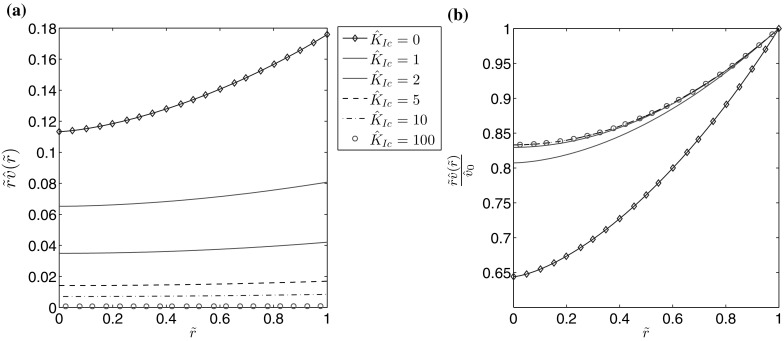
The fluid velocity for $n=0.5$ for different values of the fracture toughness: **a** the self-similar fluid velocity, **b** the self-similar fluid velocity divided by the leading term of the crack tip asymptotics (37)

**Fig. 11:**
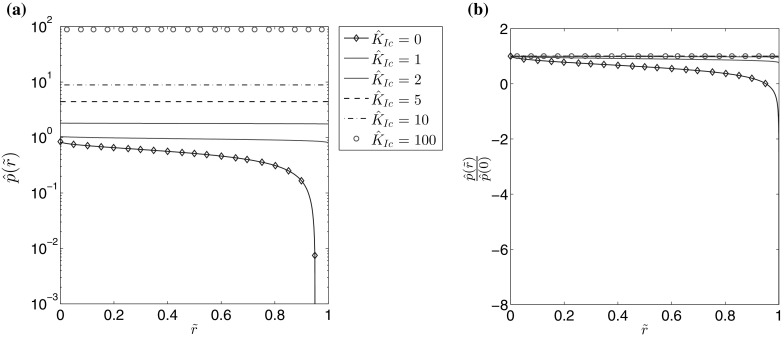
The pressure function for $n=0.5$ for different values of the fracture toughness: **a** the self-similar pressure function, **b** the self-similar pressure divided by the value of the pressure at the fracture opening

**Fig. 12:**
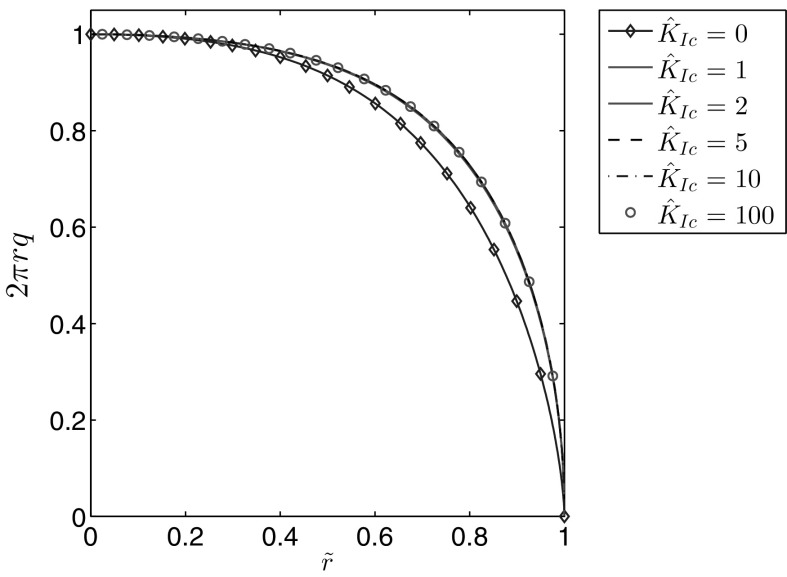
The self-similar fluid flow rate for $n=0.5$ for different values of the fracture toughness

As can be seen, over the analyzed range of *N*, the computations are very accurate and converge rapidly as the mesh density is increased. In the viscosity dominated regime it can be seen that there is a lower sensitivity of $e_w$ and $e_v$ to the value of *n*, however even in the toughness dominated mode the dependence of $e_w$ on the fluid behaviour index becomes less pronounced as $\hat{K}_{Ic}$ grows. A general trend can be observed, in that the convergence rate is magnified as the self-similar material toughness $\hat{K}_{Ic}$ increases. This is due to the fact that, for large values of $\hat{K}_{Ic}$, the solution tends to the limiting case of a uniformly pressurized immobile crack with a parabolic profile. To explain this tendency we present in Figs. 9, 10, 11 and 12 some additional data for a single value of the fluid behavior index ($n=0.5$).

**Fig. 13:**
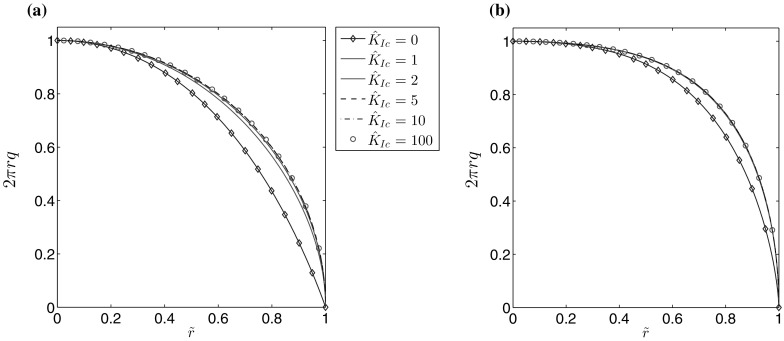
The self-similar fluid flow rate for different values of the fracture toughness when the fluid behaviour index is: **a** $\hbox {n}=0$ and **b** $\hbox {n}=1$

It is immediately obvious that for $\hat{K}_{Ic}>2$ the fracture aperture is almost entirely described by the leading term of its crack tip asymptotics (for $\hat{K}_{Ic}=2$ the maximal deviation between them is approximately 1 percent). For the fluid velocity it can be seen that, while the effect is not as substantial as for the aperture, the crack propagation speed $\hat{v}_0$ does become a better predictor of the parameter’s behaviour for larger values of the material toughness. Meanwhile, the fluid pressure increases with growing $\hat{K}_{Ic}$, eventually becoming uniformly distributed over $\tilde{r}$. As a result of the decreasing pressure gradient the velocity of the fluid flow is reduced. In Fig. 12 it can be seen that the fluid flow rate rapidly converges to the limiting case with growing $\hat{K}_{Ic}$, however the rate of convergence is greater for larger values of *n*. Indeed, as can be seen in Fig. 13, for $n=1$ the curves for $\hat{K}_{Ic}=1$ and $\hat{K}_{Ic}=100$ are indistinguishable, which is not the case when $n=0$.

In fact, the behaviour of the solution as $\hat{K}_{Ic}\rightarrow \infty$ can easily be shown to take the form:

75 $$\begin{aligned} \hat{w}(\tilde{r})\sim & {} \frac{4}{\sqrt{\pi }}\hat{K}_I \sqrt{1-\tilde{r}^2} , \quad \hat{p}(\tilde{r}) \sim \frac{\sqrt{\pi }}{2}\hat{K}_I , \quad \hat{v}_0 \sim \frac{3}{8\sqrt{\pi }\hat{K}_I(3-\rho )}, \end{aligned}$$

76 $$\begin{aligned} \tilde{r}\hat{v}(\tilde{r})= & {} \hat{v}_0 \left[ \tilde{r}^2 + \frac{3-\rho }{3} \left( 1-\tilde{r}^2\right) \right] + O\left( \hat{K}_{Ic}^{-1}\right) , \end{aligned}$$

77 $$\begin{aligned} \tilde{r}\hat{q}(\tilde{r})= & {} \frac{\sqrt{1-\tilde{r}^2}}{2\pi }\left[ \frac{3\tilde{r}^2}{3-\rho } + \left( 1-\tilde{r}^2\right) \right] + O\left( \hat{K}_{Ic}^{-1}\right) , \end{aligned}$$

where $\rho$ is defined in Table 2. As a result the computations become far more efficient in this case and the resulting solution is calculated to a far higher level of accuracy.

Combining the results shown above in Figs. 1, 2, 3, 4, 5, 6, 7 and 8, it is clear that the computations presented here achieve a very high level of accuracy for both the aperture and fluid velocity regardless of the crack propagation regime. When using $N=300$ the accuracy of computations can almost always be assumed to be correct to a level of at least $10^{-7}$ for the fracture aperture, and $2.5\times 10^{-7}$ for the fluid velocity. In this way the obtained data constitutes a very convenient and credible reference solution when testing other computational schemes. Simple (based on elementary functions) and accurate approximations of the results, which facilitates their application as benchmark data, are provided in the second part of this paper.

It is worth mentioning that the efficiency of computations achieved by this algorithm means that this high level of accuracy does not come at the expense of simulation time. The final algorithm requires fewer than 20 iterations to produce a solution. Simulation times are also very short with this scheme.

## Conclusions

In this paper, the problem of a penny-shaped hydraulic fracture driven by a power-law fluid has been analyzed. Following an approach similar to that in [24, 25] the governing equations have been reformulated in terms of the aperture *w* and the reduced fluid velocity $\Phi$. Self-similar formulations have been derived for two types of time dependent function. A computational scheme based on the universal algorithm introduced in [24] has been constructed. The accuracy of computations has been verified against a set of newly introduced analytical benchmark examples. Alternative measures of the solution accuracy have been proposed and investigated. The ability to obtain highly accurate numerical reference solutions has been demonstrated.

The following conclusions can be drawn from the conducted research.

- The universal algorithm for numerically simulating hydraulic fractures, introduced in [24], can be successfully adapted to the case of a penny-shaped fracture. It enables accurate and efficient modelling of HFs driven by the power-law fluids in both the viscosity and toughness dominated regimes.
- The key elements of the algorithm, which contributed to its outstanding performance, are: i) choice of proper computational variables, including the reduced fluid velocity, ii) extensive utilization of the information on the solution asymptotics, combined with a fracture front tracing mechanism based on the Stefan-type condition (speed equation), iii) application of the modified form of the elasticity operator (26), which has a non-singular kernel, that can easily be coupled with the new dependent variable - the reduced fluid velocity.
- The newly introduced analytical benchmark solutions, with a predefined non-zero fluid leak-off, can be adjusted to mimic the HF behaviour for a class of power-law fluids in both the viscosity and toughness dominated regimes. These solutions can be directly applied to investigate the actual error of computations when testing various computational schemes.
- The error measures $e_w$ and $e_v$ (74), based on the rate of solution convergence, have been shown to be equivalent and credible error measures for analyzing the problem when no closed-form analytical solutions are available.

While this work has allowed for the creation of highly accurate numerical benchmarks, they are not in a form which can be easily utilized. In the second part of this paper, approximate formulae for the case of an impermeable solid, constituting a set of accurate and easily accessible reference solutions when investigating other computational algorithms, will be delivered. Additionally, a brief comparison with alternative benchmarks available in the literature will be performed.

### Electronic supplementary material

Below is the link to the electronic supplementary material.

Supplementary material 1 (pdf 53 KB)

## Appendix 1: Limiting cases: Newtonian and plastic fluids

### Newtonian fluid: $n=1$

In the case of a Newtonian fluid the majority of the results remains the same as in the general case (setting $n=1$), but a few constants and functions will take alternate forms. These are detailed below.

The crack tip asymptotics in the viscosity dominated regime can be described by general relations (35)–(37). However, in the toughness dominated mode one has:

78 $$\begin{aligned} \tilde{w}(\tilde{r},\tilde{t})= & {} \tilde{w}_0(\tilde{t}) \sqrt{1-\tilde{r}^2 } + \tilde{w}_1(\tilde{t})\left( 1-\tilde{r}^2 \right) + \tilde{w}_2(\tilde{t})\left( 1-\tilde{r}^2 \right) ^{\frac{3}{2}}\log \left( 1-\tilde{r}^2 \right) \nonumber \\&+ O\left( \left( 1-\tilde{r}^2 \right) ^{\frac{3}{2}}\right) , \quad \tilde{r} \rightarrow 1, \end{aligned}$$

79 $$\begin{aligned} \frac{\partial \tilde{p}}{\partial \tilde{r}}= & {} \,\tilde{p}_0 (\tilde{t}) \left( 1-\tilde{r}^2\right) ^{-1} + \tilde{p}_1 (\tilde{t}) \left( 1-\tilde{r}^2\right) ^{-\frac{1}{2}}+ \,O\left( 1 \right) , \quad \tilde{r}\rightarrow 1 . \end{aligned}$$

The respective asymptotic expansions at the crack inlet, for both the viscosity and toughness dominated regimes, yield:

80 $$\begin{aligned} \tilde{w}(\tilde{r},\tilde{t})= & {} \tilde{w}_0^o +\tilde{w}_1^o \tilde{r} + O\left( \tilde{r}^2 \log (\tilde{r})\right) , \quad \tilde{r}\rightarrow 0 , \end{aligned}$$

81 $$\begin{aligned} \tilde{p}(\tilde{r},\tilde{t})= & {} \tilde{p}_0^o (\tilde{t}) + \tilde{p}_1^o (\tilde{t}) \log \left( \tilde{r}\right) + O\left( \tilde{r}\right) , \quad \tilde{r}\rightarrow 0 . \end{aligned}$$

It should be noted that the pressure is singular at the fracture origin, which is not the case for non-Newtonian ($n<1$) fluids.

Meanwhile, the relationship between the new variable $\Omega$ and the pressure, in the time-dependent formulation, follows from the definition (44):

82 $$\begin{aligned} \tilde{p}(\tilde{r},\tilde{t})= \Omega _0(\tilde{t})\log (\tilde{r})+C_p(\tilde{t})+\int _0^{\tilde{r}}\Omega (\xi , \tilde{t})d\xi , \end{aligned}$$

where the time dependent constant $C_p(\tilde{t})$ is obtained by expanding (25) using (44):

83 $$\begin{aligned} C_p (\tilde{t}) = \frac{1}{2}\sqrt{\frac{\pi }{L(\tilde{t})}} \tilde{K}_I + \left[ 1 -\log \left( 2\right) \right] \Omega _0 (\tilde{t}) - \int _0^1 \Omega (y ,\tilde{t} )\sqrt{1-y^2} \, dy . \end{aligned}$$

Transforming into the self-similar formulation (54), these become:

84 $$\begin{aligned} \hat{p}(\tilde{r})= & {} \,\hat{\Omega }_0\log \left( \tilde{r}\right) + \hat{C}_p+\int _0^{\tilde{r}} \hat{\Omega }(\xi ) \, d\xi , \end{aligned}$$

85 $$\begin{aligned} \hat{C}_p= & {} \frac{\sqrt{\pi }}{2} \hat{K}_I + \left[ 1 -\log \left( 2\right) \right] \hat{\Omega }_0 - \int _0^1 \hat{\Omega }(y)\sqrt{1-y^2} \, dy . \end{aligned}$$

Finally, the auxiliary function ${\mathcal {G}}_n(\tilde{r})$ will now be expressed as:

86 $$\begin{aligned} {\mathcal {G}}_n (\tilde{r}) = \tilde{r}\left( \frac{\pi }{2} - \arctan \left( \frac{\tilde{r}}{\sqrt{1-\tilde{r}^2}}\right) \right) - \sqrt{1-\tilde{r}^2} \equiv \tilde{r}\arccos \left( \tilde{r}\right) - \sqrt{1-\tilde{r}^2} . \end{aligned}$$

### Perfectly plastic fluid: $n=0$

In the case of a perfectly plastic fluid, alongside changes to the system asymptotics and reformulated equations, the degeneration of the Poiseuille equation means that it cannot be used to define the fluid velocity $\tilde{v}$, or the reduced fluid velocity $\Phi$. As a result fundamental changes to the scheme are required. These are outlined below.

The crack tip asymptotics in the viscosity dominated regime remains in the same form as was outlined in (35)–(37). In the toughness dominated mode however it now yields:

87 $$\begin{aligned} \tilde{w}(\tilde{r},\tilde{t})= & {}\, \tilde{w}_0(\tilde{t}) \sqrt{1-\tilde{r}^2 }+ \tilde{w}_1(\tilde{t})\left( 1-\tilde{r}^2 \right) ^{\frac{3}{2}}\log \left( 1-\tilde{r}^2 \right) + \tilde{w}_2(\tilde{t})\left( 1-\tilde{r}^2 \right) ^{\frac{3}{2}} \nonumber \\&+ O\left( \left( 1-\tilde{r}^2 \right) ^{\frac{5}{2}}\right) , \quad \tilde{r} \rightarrow 1, \end{aligned}$$

88 $$\begin{aligned} \frac{\partial \tilde{p}}{\partial \tilde{r}}= & {}\, \tilde{p}_0 (\tilde{t}) \left( 1-\tilde{r}^2\right) ^{-\frac{1}{2}} + O\left( 1 \right) , \quad \tilde{r}\rightarrow 1 . \end{aligned}$$

The fracture opening and the fluid pressure can be estimated at the crack inlet as:

89 $$\begin{aligned} \tilde{w}(\tilde{r},\tilde{t})= & {} \tilde{w}_0^o (\tilde{t})+ O\left( \tilde{r}^2 \log (\tilde{r})\right) , \quad \tilde{r}\rightarrow 0 , \end{aligned}$$

90 $$\begin{aligned} \tilde{p}(\tilde{r},\tilde{t})= & {} \tilde{p}_0^o (\tilde{t}) + \tilde{p}_1^o (\tilde{t}) \tilde{r} + O\left( \tilde{r}^2 \right) , \quad \tilde{r}\rightarrow 0 . \end{aligned}$$

Meanwhile, the relationship between the modified fluid pressure derivative and the pressure follows from the definition (44):

91 $$\begin{aligned} \tilde{p}(\tilde{r},\tilde{t}) = \tilde{r} \Omega _0 (\tilde{t} ) + C_p (\tilde{t}) + \int _0^{\tilde{r}} \Omega ( \xi , \tilde{t}) \, d\xi , \end{aligned}$$

where

92 $$\begin{aligned} C_p = \frac{1}{2}\sqrt{\frac{\pi }{L(\tilde{t})}} \tilde{K}_I - \frac{\pi }{4}\Omega _0 (\tilde{t}) - \int _0^{1} \hat{\Omega }(y, \tilde{t})\sqrt{1-y^2} \, dy . \end{aligned}$$

Note, from the form of the above, that the pressure is not singular at the injection point in this case. Transforming into the self-similar formulation (54) these become:

93 $$\begin{aligned} \hat{p}(\tilde{r})= & {}\, \tilde{r}\hat{\Omega }_0 + \hat{C}_p + \int _0^{\tilde{r}} \Omega (\xi ) \, d\xi , \end{aligned}$$

94 $$\begin{aligned} \hat{C}_p= & {} \frac{\sqrt{\pi }}{2}\hat{K}_I - \frac{\pi }{4}\hat{\Omega }_0 - \int _0^{1} \hat{\Omega }(y)\sqrt{1-y^2} \, dy . \end{aligned}$$

It can be shown that the relationship between $\Omega$ and the fracture aperture (52) still holds, with the function ${\mathcal {G}}_n (\tilde{r})$ being given by:

95 $$\begin{aligned} \begin{aligned} {\mathcal {G}}_n (\tilde{r})&= -\frac{\pi }{8} \left[ \sqrt{1-\tilde{r}^2} + \tilde{r}^2 \log \left( \frac{\tilde{r}}{1+\sqrt{1-\tilde{r}^2}}\right) \right]&\\&\equiv -\frac{\pi }{8} \left[ \sqrt{1-\tilde{r}^2} - \tilde{r}^2 {{\mathrm{arctanh}}}\left( \sqrt{1-\tilde{r}^2} \right) \right] .&\end{aligned} \end{aligned}$$

In practice however, the degeneration of the Poiseuille equation means that a new scheme for solving the governing equations must be devised. The first step towards this is to note that the fracture aperture can be expressed as a non-linear integral equation:

96 $$\begin{aligned} \hat{w} (\tilde{r}) = -\frac{8}{\pi }\int _0^1 \frac{1}{\hat{w}(y)} {\mathcal {K}}(y,\tilde{r}) , dy + \frac{4}{\sqrt{\pi }} \hat{K}_I \sqrt{1-\tilde{r}^2} , \end{aligned}$$

while the crack-propagation speed is calculated from the fluid balance Eq. (68) as follows:

97 $$\begin{aligned} \hat{v}_0 = \frac{\hat{Q}_0}{2\pi \left[ \left( 3-\rho \right) \int _0^1 \tilde{r}\hat{w}(\tilde{r}) \, d\tilde{r} + \frac{1-\rho }{\gamma }\int _0^1 \tilde{r} \hat{q}_l \, d\tilde{r}\right] } . \end{aligned}$$

The reduced fluid velocity $\hat{\Phi }$ can be determined by integrating (67):

98 $$\begin{aligned} \hat{\Phi }(\tilde{r}) = \frac{\hat{v}_0}{\hat{w}(\tilde{r})} \int _{\tilde{r}}^1 \xi \left[ \left( 3-\rho \right) \hat{w}(\xi )+\left( 1-\rho \right) \frac{\hat{q}_l(\xi )}{\gamma } \right] \, d\xi . \end{aligned}$$

Relations (96)–(98) are embedded accordingly in the general numerical scheme.
