# Supplementary material for: Fluid velocity based simulation of hydraulic fracture: a penny shaped model—part I: the numerical algorithm
Source: Meccanica. 2018 Oct 22;53(15):3615–35. doi: 10.1007/s11012-018-0899-y (PMC6405075; doi:10.1007/s11012-018-0899-y)
Supplement: Supplementary file 1 — Supplementary material 1 (pdf 53 KB) [file 11012_2018_899_MOESM1_ESM.pdf]

Fluid velocity based simulation of hydraulic fracture - a penny shaped model. Part I: the numerical algorithm
Supplementary material

D. Peck, M. Wrobel, M. Perkowska, G. Mishuris

S1 Analytical benchmarks

In the following we will present a way to construct a set of analytical benchmark solutions that satisfy the system of governing equations (61)-(70) for the self-similar problem. Those solutions can be easily extended through the relations (19)-(21), (25), (29) and (51)-(52) to the time dependent forms. In this way one can formulate a set of analytical benchmark examples for both, the self-similar and the time dependent versions of the problem.

The basic concept employed to derive the self-similar solutions is the same as that in [24] for the KGD model. We assume that the crack aperture can be expressed as a weighted sum of properly chosen base functions:

$$\hat{w}(\tilde{r}) = \sum_{i=0}^{M} \lambda_i h_i(\tilde{r}). \tag{S1}$$

The functions h_i are selected in a way that enables one to: i) comply with the asymptotic representation (35), ii) satisfy the respective boundary conditions (70), iii) compute analytically the elasticity operator (63). The multipliers λ_i are to be chosen properly to ensure the physically justified behaviour and desired properties of the solution.

Provided that iii) is satisfied, the fluid pressure function can be computed in a closed form from (63) to give:

$$\hat{p}(\tilde{r}) = \sum_{i=0}^{M} \lambda_i \pi_i(\tilde{r}), \tag{S2}$$

where each function π_i corresponds to respective function h_i.

The self-similar stress intensity factor follows immediately from the asymptotic behaviour of functions h_i and complies with (69). Next, the self-similar crack propagation speed, $\hat{v}_0$ can be determined according to (61), while the fluid velocity is computed from (66) to produce:

$$\hat{v}(\tilde{r}) = \left\{ - \left[\sum_{i=0}^{M} \lambda_i h_i(\tilde{r}) \right]^{n+1} \cdot \sum_{i=0}^{M} \lambda_i \pi_i'(\tilde{r}) \right\}^{1/n}. \tag{S3}$$

Consequently, the reduced fluid velocity is defined by employing (S2) in $(57)_1$. The influx magnitude, $\hat{Q}_0$, is computed from (70), while the modified fluid pressure derivative can be obtained from the definition $(57)_2$, (59). Finally, the benchmark leak-off function is determined by a transformation of (67) as:

$$\hat{q}_l(\tilde{r}) = \frac{\gamma}{1-\rho} \left[(\rho - 3)\hat{w}(\tilde{r}) - \frac{1}{r\hat{v}_0} \left(\hat{w}(\tilde{r}) \hat{\phi}(\tilde{r}) \right)' \right], \tag{S4}$$

where the quantities on the right hand side are taken according to (S1)-(S3).

In this way, by using different values of the coefficients λ_i and different functions $h_i(\tilde{r})$, $\pi_i(\tilde{r})$ one can construct a number of self-similar problems for various fluid behaviour indices and crack propagation regimes, for which there exist known purely analytical solutions in the form (S1)-(S3). The values of pumping rate, $\hat{Q}_0$, and the self-similar material toughness, $\hat{K}_{Ic}$, can be tuned by the choice of magnitudes of respective coefficients λ_i.

The examples of base functions $h_i(\tilde{r})$, $\pi_i(\tilde{r})$ are collected in Table S1.

i	$\pi_i(\tilde{r})$	$h_i(\tilde{r})$
1	1	$\frac{8}{\pi}\sqrt{1-\tilde{r}^2}$
2	$\tilde{r}$	$\sqrt{1-\tilde{r}^2}+\tilde{r}^2\log\left(\frac{1+\sqrt{1-\tilde{r}^2}}{\tilde{r}}\right)$
3	$\tilde{r}^{1-n}$	$\frac{2\Gamma\left(\frac{3}{2}-\frac{n}{2}\right)\Gamma\left(\frac{n}{2}-1\right)}{\Gamma\left(2-\frac{n}{2}\right)\Gamma\left(\frac{n}{2}-\frac{1}{2}\right)}\left[\tilde{r}^{2-n}-\frac{\Gamma\left(\frac{n}{2}-\frac{1}{2}\right)}{\sqrt{\pi}\Gamma\left(\frac{n}{2}\right)}{}_2F_1\left(\frac{1}{2},\frac{n}{2}-1;\frac{n}{2};\tilde{r}^2\right)\right]$
4	$\tilde{r}^{2-n}$	$\frac{2\Gamma\left(2-\frac{n}{2}\right)\Gamma\left(\frac{n-3}{2}\right)}{\Gamma\left(\frac{5-n}{2}\right)\Gamma\left(\frac{n}{2}-1\right)}\left[\tilde{r}^{3-n}-\frac{\Gamma\left(\frac{n}{2}-1\right)}{\sqrt{\pi}\Gamma\left(\frac{n-1}{2}\right)}{}_2F_1\left(\frac{1}{2},\frac{n-3}{2};\frac{n-1}{2};\tilde{r}^2\right)\right]$
5	$\log(\tilde{r})$	$\frac{8}{\pi}\left[\tilde{r}\arccos\left(\tilde{r}\right)+\left(\log(2)-2\right)\sqrt{1-\tilde{r}^2}\right]$
6	$\tilde{r}{}_2F_1\left(\frac{1}{2}-\alpha,1;\frac{1}{2};\tilde{r}^2\right)$	$\frac{2\sqrt{\pi}\left(1-\tilde{r}^2\right)^\alpha}{1+2\alpha}\left[\frac{\Gamma\left(\alpha+\frac{1}{2}\right)}{\Gamma(1+\alpha)}{}_2F_1\left(\frac{1}{2},\frac{1}{2}+\alpha;1+\alpha;1-\tilde{r}^2\right)+\frac{4\Gamma\left(\frac{3}{2}+\alpha\right)}{(1+2\alpha)\Gamma(\alpha)}{}_2F_1\left(-\frac{1}{2},\frac{1}{2}+\alpha;1+\alpha;1-\tilde{r}^2\right)\right]-\frac{4}{1+2\alpha}\log\left(\frac{1+\sqrt{1-\tilde{r}^2}}{\tilde{r}}\right)$
7	$\mathrm{arctanh}(\tilde{r})$	$4\left[E\left(1-\tilde{r}^2\right)-K\left(1-\tilde{r}^2\right)+\log\left(\frac{1+\sqrt{1-\tilde{r}^2}}{\tilde{r}}\right)\right]$

Table S1: Table showing the components of the benchmark solutions. Here ${}_2F_1$ is the Gaussian hypergeometric function, while functions K, E represent the complete elliptic integral of the first and second kinds respectively.

To provide a very simple example of a numerical benchmarks which can be created using the aforementioned methodology, we consider the following composite functions:

$$h_A(\tilde{r},\alpha)=h_6(\tilde{r},\alpha)+\frac{\pi}{1+2\alpha}h_1-\frac{2}{1+2\alpha}h_2(\tilde{r}), \tag{S5}$$

$$h_B(\tilde{r},n)=-h_3(\tilde{r},n)+\frac{n\sqrt{\pi}\Gamma\left(\frac{3-n}{2}\right)}{2\Gamma\left(2-\frac{n}{2}\right)}h_1+\frac{2\left(1-n\right)\Gamma\left(\frac{3-n}{2}\right)}{\sqrt{\pi}\Gamma\left(2-\frac{n}{2}\right)}h_2(\tilde{r}), \tag{S6}$$

with the corresponding pressure terms:

$$\pi_A(\tilde{r},\alpha) = \pi_6(\tilde{r},\alpha) + \frac{\pi}{1+2\alpha}\pi_1 - \frac{2}{1+2\alpha}\pi_2(\tilde{r}), \tag{S7}$$

$$\pi_B(\tilde{r},n) = -\pi_3(\tilde{r},n) + \frac{n\sqrt{\pi}\Gamma\left(\frac{3-n}{2}\right)}{2\Gamma\left(2-\frac{n}{2}\right)}\pi_1 + \frac{2\left(1-n\right)\Gamma\left(\frac{3-n}{2}\right)}{\sqrt{\pi}\Gamma\left(2-\frac{n}{2}\right)}\pi_2(\tilde{r}). \tag{S8}$$

Then the asymptotic behaviour of the respective functions at the fracture tip is:

$$h_A(\tilde{r},\alpha) = \frac{2\sqrt{\pi}\Gamma\left(\alpha+\frac{1}{2}\right)}{\Gamma\left(\alpha+1\right)}\left(1-\tilde{r}^2\right)^{\alpha} + O\left(\left(1-\tilde{r}^2\right)^{\min\left(\frac{5}{2},\alpha+1\right)}\right), \quad \tilde{r}\to 1, \tag{S9}$$

$$\frac{d\pi_A(\tilde{r},\alpha)}{d\tilde{r}} = \frac{\sqrt{\pi}\left(1-2\alpha\right)\Gamma\left(2-\alpha\right)}{\Gamma\left(\frac{3}{2}-\alpha\right)}\left(1-\tilde{r}^2\right)^{\alpha-2} + O\left(\left(1-\tilde{r}^2\right)^{\alpha-1}\right), \quad \tilde{r}\to 1, \tag{S10}$$

$$h_B(\tilde{r},n) = O\left(\left(1-\tilde{r}^2\right)^{\frac{5}{2}}\right), \quad \tilde{r}\to 1, \tag{S11}$$

$$\frac{d\pi_B(\tilde{r},n)}{d\tilde{r}} = \left(1-n\right)\left[\frac{2\Gamma\left(\frac{3-n}{2}\right)}{\sqrt{\pi}\Gamma\left(2-\frac{n}{2}\right)} - 1\right] + O\left(1-\tilde{r}^2\right), \quad \tilde{r}\to 1, \tag{S12}$$

It can easily be seen from the above equations that the functions h_A and π_A will provide the proper first term of the crack tip asymptotics for the aperture (35) and pressure derivative (36), (41), provided that α is taken in accordance with Table 1. Further terms may also be constructed, although subsequent (known) asymptotic terms of h_A and π_A must be accounted for. Additionally the behaviour of h_B, π_B at the crack tip ensures that it will not interfere with the final asymptotics of the benchmark at the fracture front in a notable way.

Meanwhile, at the crack inlet, we have:

$$h_A(\tilde{r},\alpha) = \frac{2}{1+2\alpha}\left[3 + \frac{4\alpha}{1+2\alpha} - H\left(\alpha - \frac{1}{2}\right)\right] + O(\tilde{r}^2\log(\tilde{r})), \quad \tilde{r}\to 0, \tag{S13}$$

$$\frac{d\pi_A(\tilde{r},\alpha)}{d\tilde{r}} = \frac{2\alpha-1}{1+2\alpha} + O(\tilde{r}^2), \quad \tilde{r}\to 0, \tag{S14}$$

$$h_B(\tilde{r},n) = -\frac{2n\sqrt{\pi}\left(1-n\right)\sec\left(\frac{n\pi}{2}\right)}{\left(2-n\right)\Gamma\left(2-\frac{n}{2}\right)\Gamma\left(\frac{n-1}{2}\right)} + O(\tilde{r}^{2-n}), \quad \tilde{r}\to 0, \tag{S15}$$

$$\frac{d\pi_B(\tilde{r},n)}{d\tilde{r}} = -\left(1-n\right)\tilde{r}^{-n} + O(1), \quad \tilde{r}\to 0 \tag{S16}$$

where H is the harmonic number function and α can be taken in accordance with Table 1. From this it can be easily seen that the required asymptotic representations of the aperture (34) and pressure derivative (32) will be satisfied by h_B and π_B, while the fracture opening asymptotics of h_A and π_A will not prevent the benchmark from displaying the correct behaviour. As with the crack tip, here further asymptotic terms can be accounted for using additional functions.

In this way, by linear combination of functions (S5)–(S8) and other functions from Table S1 one can build a benchmark example for the viscosity dominated regime of crack propagation for a number of shear-thinning fluids, provided that $\alpha = \alpha_0$. Moreover, by incorporation of function h_0 from Table S1 we obtain a solution which mimics the toughness dominated mode.

The above strategy have been successfully employed to create a set of analytical benchmark examples for the the varying crack propagation regimes and fluid behaviour indices.
